# Overexpression of SENP3 in oral squamous cell carcinoma and its association with differentiation

**DOI:** 10.3892/or.2013.2318

**Published:** 2013-03-01

**Authors:** ZUJUN SUN, SHUIQING HU, QINGQIONG LUO, DONGXIA YE, DAN HU, FUXIANG CHEN

**Affiliations:** 1 Department of Clinical Laboratories, Ninth People’s Hospital, Shanghai Jiao Tong University School of Medicine, Shanghai 200011, P.R. China; 2 Department of Oral and Maxillofacial Surgery, Ninth People’s Hospital, Shanghai Jiao Tong University School of Medicine, Shanghai 200011, P.R. China

**Keywords:** small ubiquitin-like modifier, small ubiquitin-like modifier-specific proteases, oral squamous cell carcinoma, reactive oxygen species

## Abstract

Small ubiquitin-like modifier (SUMO) modification is an important post-translational protein modification that can be reversed by SUMO-specific proteases (SENPs); however, the physiological function of SENPs remains largely unexplored, and little is known about the regulation of SENPs themselves. As one of the crucial members of the SUMO system, SENP3 is essential for rRNA processing and cell proliferation. In the present study, we analysed the expression of SENP3 in human oral squamous cell carcinoma (OSCC) and investigated the correlation between its expression and clinicopathological parameters in OSCC patients. The expression of SENP3 was higher in OSCC tissues than that in the normal mucosa adjacent to the tumor, and a modest increase in reactive oxygen species (ROS) regulated SENP3 stability and localization. ROS induced SENP3 redistribution from the nucleoli to the nucleoplasm. Taken together, these results indicated that the expression level of SENP3 may be associated with the differentiation of OSCC and that SENP3 may play an important role in the development of OSCC under oxidative stress.

## Introduction

Oral squamous cell carcinoma (OSCC) is one of the most common malignant tumors in Southeast Asia, afflicting approximately 300,000 patients worldwide each year (1,2). The 5-year survival rate of OSCC patients is approximately 50–60%, and the rate is even lower in patients diagnosed at later stages (3,4). However, the mechanisms involved in the tumorigenesis of OSCC have not yet been well characterised.

Small ubiquitin-like modifier (SUMO) modification (SUMOylation) is an important post-translational protein modification that has gained much prominence due to the large number of SUMO substrates (5). SUMOylation is catalysed by SUMO-specific E1, E2 and E3 enzymes, and the covalent modification of proteins is reversed by a family of Sentrin/SUMO-specific proteases (SENPs) (6). SUMOylation can regulate a broad spectrum of cellular processes, including DNA replication/repair, cell division, cell signal transduction and nuclear transportation. De-SUMOylation mediated by SENPs has been shown to play an important role in these processes as well (7–12). SENPs deconjugate modified proteins and are thus critical for maintaining the level of SUMOylated and un-SUMOylated substrates required for normal physiology. The altered expression of SENPs has been observed in several carcinomas. SENP1 can transform normal prostate epithelia into a dysplastic state and directly modulate several oncogenic pathways in prostate cells, including the androgen receptor, c-Jun and cyclin D1 pathways (8,13). The assessment of tissues from human prostate cancer patients has revealed elevated mRNA levels of SENP1 and SENP3 (13). As one of the essential members of the SENP family, SENP3 is located in the nucleolus and appears to have a distinct preference for deconjugating SUMO2/3 (11,14). The induction of SENP3 in cancer cells initiates the angiogenic pathway, and SENP3 regulates the transcriptional activity of hypoxia-inducible factor 1α (HIF1α) via de-SUMOylation of the co-regulatory protein, p300 (15). SENP3 expression is also increased in prostate cancer and other carcinomas including, ovarian, lung, rectal and colon carcinomas (16). However, the mechanisms which trigger de-SUMOylation and regulate SENP3 under various physiological and pathological conditions have not yet been elucidated, and the functions and related mechanisms of SENP3 in OSCC remain unknown.

It has previously been reported that SUMO conjugation, in particular SUMO2/3 conjugation, is a major response to oxidative stress, and the balance between SUMOylation and de-SUMOylation may play a critical role in the cellular adaptive response to reactive oxygen species (ROS) production (17–22). A number of studies have found that the development of OSCC correlates with oxidative stress (23,24). It is unknown, however, whether the SUMO2/3-specific protease, SENP3, is involved in OSCC development in response to oxidative stress.

To date, research on SUMOylation related to OSCC are limited. Therefore, the aim of this study was to investigate the expression of SENP3 in OSCC cell lines and clinical tissue samples from OSCC patients, as well as the possible correlations between SENP3 expression and the clinicopathological characteristics of OSCC patients.

## Materials and methods

### Cell lines and reagents

The HIOEC cell line and the human OSCC cell lines, HB, CAL-27, WSU-HN6, SCC-4, SCC-9, SCC-25 and LEUK-1, were obtained from the Laboratory of Oral Oncology, Ninth People’s Hospital, School of Medicine, Shanghai Jiao Tong University. HIOEC cells were maintained in defined keratinocyte-SFM medium (Gibco-BRL, Carlsbad, CA, USA) and the other cells were maintained in DMEM supplemented with 10% heat-inactivated fetal bovine serum (Gibco). All cells were cultured in a humidified atmosphere of 5% CO_2_ at 37°C. To investigate the association with ROS, cells were treated with H_2_O_2_ and N-acetyl cysteine (NAC) (Gibco). RFP-SENP3 was constructed by our laboratory.

### Specimens and immunohistochemistry

Forty human OSCC specimens were collected from patients who had undergone surgery between September 2009 and September 2010 in the Department of Oral and Maxillofacial Surgery, Ninth People’s Hospital, School of Medicine, Shanghai Jiao Tong University, Shanghai, China. All experimental procedures received ethics approval from the Independent Ethics Committee of Shanghai Ninth People’s Hospital affiliated to Shanghai Jiao Tong University School of Medicine (no. 200926). The pathological characterization of the OSCC patients included in this study is summarized in Table II. For immunohistochemical examination, OSCC tissues were fixed with 4% paraformaldehyde and embedded with paraffin. Sections of the samples were blocked with 10% goat serum in PBS and incubated overnight at 4°C with anti-SENP3 antibody (Abcam). After 3 washes with PBS, the sections were incubated with peroxidase-conjugated goat anti-rabbit antibody for 1 h, followed by incubation with 3,3′-diaminobenzidine (DAB) substrate for 3 min. Counter-staining was performed with hematoxylin, and dehydration was then performed with ethanol and dimethyl benzene. Slides were mounted with Permount (Santa Cruz Biotechnology, Inc.), and visualised under a light microscope (Axio Imager; Carl Zeiss, Jena, Germany). The histological grade of the tumors was determined according to the degree of differentiation in the WHO histological criteria (25).

### Reverse transcription-PCR (RT-PCR)

To determine SENP3 expression at the mRNA level, RT-PCR was performed. Total RNA was isolated using TRIzol reagent (Invitrogen, San Diego, USA) and the reverse transcription of cDNA was performed using an Omniscript RT kit (Promega) according to the manufacturer’s instructions. Primer sequences used were as follows: SENP3, 5′-GGCAGAATAATGACAGTGAC-3′ (forward) and 5′-AGTGACACAGCTCCTTGT-3′ (reverse); GAPDH, 5′-CTC CTCCACATCCCTTCC-3′ (forward) and 5′-CCGCACGT TCAAGAACAGAGA-3′ (reverse). Thermocycler conditions comprised of an initial denaturation at 94°C for 5 min, 35 cycles of PCR program: denaturing at 94°C for 30 sec, annealing at 55°C for 30 sec and elongation at 72°C for 30 sec and a final extension at 72°C for 10 min. The amplified PCR products were electrophoresed on a 1.5% agarose gel and visualised with ethidium bromide. The electrophoretic bands were documented with the Gene Genius Gel Documentation System (Syngene Inc., Cambridge, UK).

### Western blot analysis

CAL-27 cells were stimulated with H_2_O_2_ at various concentrations at 37°C and then lysed with M-PER^®^ Mammalian Protein Extraction (Pierce). Proteins were quantified by the BCA Protein Assay kit (Pierce) according to the manufacturer’s instructions. Samples containing a total of 50 μg protein were incubated at 100°C for 5 min, separated by SDS-polyacrylamide gel electrophoresis, and subsequently electrotransferred onto a polyvinylidene difluoride membrane. Essential component detection in the cells was performed with anti-SENP3 (Abcam) antibody at overnight incubation at 4°C, and then HRP-conjugated secondary antibody (1:5,000 dilution; Pierce Chemical) was added for 1 h at room temperature, followed by the development of reactions in a chemiluminescent detection system. β-actin antibody was used as the control.

### Immunostaining

CAL-27 cells deposited on glass were transfected with RFP-SENP3 for 24 h with Lipofectamine 2000 (Invitrogen), and stimulated with H_2_O_2_ for 1 h. After washing, the cells were fixed with 4% paraformaldehyde in PBS for 20 min. The sections were then mounted in medium containing DAPI for 5 min to visualize cell nuclei. Slides were evaluated with a laser scanning confocal microscope (TCS SP2; Leica, Wetzlar, Germany).

### Statistical analysis

Statistical analysis was performed using SPSS 10.0 software (SPSS Inc., Chicago, IL, USA). The statistical difference of the initial data was analysed by non-parametric tests. P<0.05 was considered to indicate a statistically significant difference.

## Results

### SENP3 protein expression is higher in tumors and is associated with clinicopathological characteristics in patients with OSCC

**Figure 1:**
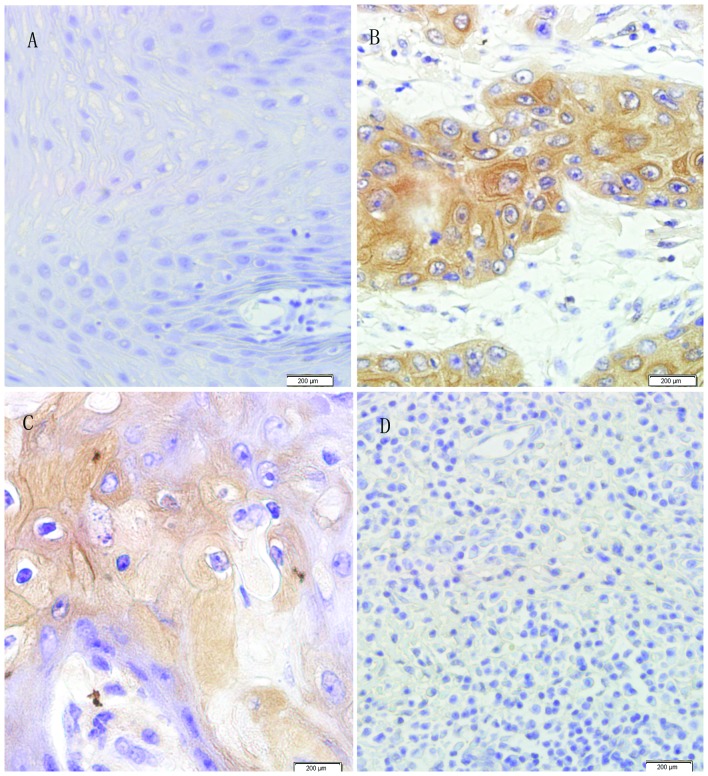
Expression of SENP3 in oral epithelial tissue adjacent to tumor and oral squamous cell carcinoma (OSCC) tissues. SENP3 expression in (A) oral epithelial tissue adjacent to tumor, (B) well-differentiated, (C) moderately differentiated and (D) poorly differentiated OSCC (×200).

The immunohistochemistry results showed that the expression of SENP3 was mainly found in the cellular cytoplasm and nucleolus (Fig. 1). Of the 40 cases, 10% (4/40) of the cases had a negative expression of SENP3, 30% (12/40) had a positive reactivity of grade 1, 25% (10/40) a positive reactivity of grade 2, and 35% (14/40) a positive reactivity of grade 3. However, oral epithelial tissue adjacent to the tumor showed negative reactivity in 30% (6/20) of the cases, a positive reactivity of grade 1 in 50% (10/20) of the cases and a positive reactivity of grade 2 in 20% (4/20) of the cases (Table I). The immunoreactions of SENP3 protein expression in the cancerous tissues were consistently stronger than those in the paired adjacent non-malignant epithelia (Wilcoxon signed-rank test Z=−2.337, P<0.05, Table I). The difference in the SENP3 protein positivity score in cancerous tissues was analysed by classification of the different clinicopathological characteristics of the OSCC patients. The protein expression level of SENP3 in the cancerous tissues was found to correlate with the pathological differentiation grade of OSCC (P<0.05). Well-differentiated cancerous tissues displayed the strongest staining (Fig. 1B), moderately differentiated cancerous tissues stained weaker (Fig. 1C) and poorly differentiated cancerous tissues displayed the weakest staining (Fig. 1D). No statistically significant associations were detected among SENP3 expression and other clinicopathological characteristics (Table II).

### H_2_O_2_ induces a rapid increase of SENP3 protein expression in OSCC cell lines

**Figure 2:**
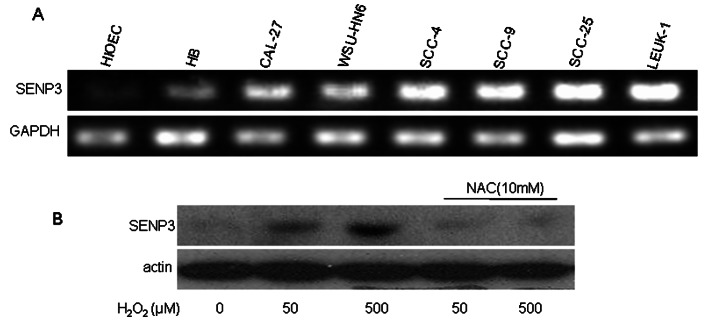
Expression of SENP3 in OSCC cell lines. H_2_O_2_ induced a rapid stabilization of SENP3 protein. (A) SENP3 mRNA levels in the human immortalised oral epithelial cells and OSCC cell lines were evaluated by RT-PCR. GAPDH was used as the internal control. (B) CAL-27 cells were pre-treated with 10 mM NAC for 4 h prior to H_2_O_2_ exposure for another 1 h. SENP3 protein levels were evaluated by immunoblotting. β-actin was used as the loading control.

SENP3 mRNA levels were higher in OSCC cell lines than in the human immortalized oral epithelial cells HIOEC (Fig. 2A). CAL-27 cells were exposed to various concentrations of H_2_O_2_ and the expression of SENP3 was evaluated. The increase of SENP3 protein expression was observed following treatment with 50 μM H_2_O_2_, and this increase occurred in a dose-dependent manner (Fig. 2B). As ROS generation is common to some stress inducers, we examined whether the increase in the SENP3 protein level could be blocked by anti-oxidants such as NAC. Indeed, the increase in SENP3 protein expression was blocked by NAC (Fig. 2B), suggesting that the content of SENP3 protein is regulated by changes in the cellular redox state.

### SENP3 is redistributed from the nucleolus to the nucleoplasm upon exposure to H_2_O_2_

**Figure 3:**
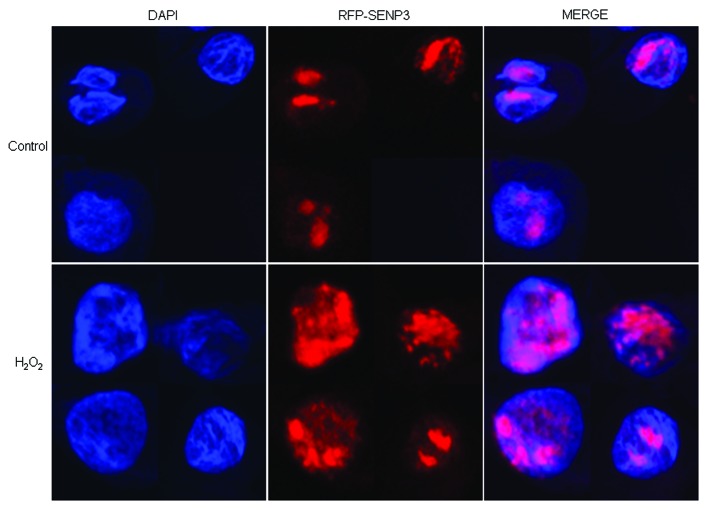
SENP3 is redistributed between the nucleolus and the nucleoplasm upon exposure to H_2_O_2_. CAL-27 cells were transfected with RFP-SENP3 for 24 h, and the cells were then treated with 100 μM H_2_O_2_ for 1 h. Cell monolayers were fixed and cell nuclei were stained with DAPI (×200).

SENP3 has been reported to be preferentially localized in the nucleolus (11,29). Therefore, we examined whether the localization of SENP3 in OSCC cells could be altered following exposure of the cells to H_2_O_2_. RFP-SENP3 was overexpressed in the CAL-27 cells and it was found to be mainly localized in the nucleolus in the untreated cells as expected, while it was almost invisible in the nucleoplasm (Fig. 3). However, SENP3 appeared in the nucleoplasm when the cells were exposed to H_2_O_2_, observed as a dim dispersion with numerous bright foci (Fig. 3), which indicated that the localization of SENP3 was actually regulated by H_2_O_2_.

## Discussion

The conjugation and de-conjugation of SUMOylation to substrates are sophisticatedly controlled and play an important physiological and pathological role in determining the outcome of numerous cellular processes. Although a number of studies have revealed the correlation between SUMOylation and tumors, there are only a few studies on SENP family expression in tumors. SENP1 is the most potent regulator of androgen receptor-dependent transcription (9). Certain studies have shown that SENP1 is associated with the development of prostate cancer and that SENP1 overexpression is found in thyroid oncocytic tumors (14,26). SENP5 is required to maintain mitochondrial morphology and function, as well as cell division (11,27). SENP5 has been shown to be expressed in OSCC and correlates with the differentiation of OSCC (28). However, the association between other members of the SENP family and OSCC has not yet been extensively investigated.

As one of the essential members of the SENP family, SENP3 is localized in the nucleolus, which suggests that it may be involved in regulating certain aspects of nucleolar function (11,29). In addition, SENP3 prefers SUMO2/3 as its substrates and is required for rRNA processing and maturation through de-conjugation of SUMO2/3 from nucleophosmin (11,12). SENP3-mediated de-conjugation of SUMO2/3 from promyelocytic leukemia has been shown to correlate with accelerated cell proliferation under mild oxidative stress (16). SENP3 is also responsible for enhancing HIF-1 transactivation under mild oxidative stress via p300 de-SUMOylation (15). The stability of SENP3 is regulated by ROS via interactions with CHIP and Hsp90 (30). However, the correlation between the overexpression of SENP3 and its association with OSCC has yet not been elucidated.

In the present study, we found that SENP3 was overexpressed in OSCC cell lines compared to HIOEC, a normal oral epithelial cell line (Fig. 2A). SENP3 protein expression was higher in OSCC tissues compared to epithelial adjacent to tumor tissues (Table I), which indicated that SENP3 was associated with OSCC differentiation. A significant correlation between SENP3 protein expression and the pathological differentiation grade (P<0.05) was observed in our study (Table II). This correlation implies that the well-differentiated cases have a higher SENP3 expression. This suggests the possibility that SENP3 may be a marker of differentiation in OSCC and has implications in the induced differentiation therapy of OSCC.

As is well known, smoking, alcohol consumption and chewing betel nut are the main factors in inducing oral squamous cell carcinogenesis, and all can produce ROS (23,24). At the same time, mild ROS can induce SENP3 stabilization and activation. SENP3 is a redox sensor, which can regulate transcription factor (TF) activity under oxidative stress through the de-SUMOylation of proteins (31). We also found that the accumulation and redistribution of SENP3 in OSCC cell lines in response to oxidative stress, may enable SENP3 to de-SUMOylate its extra-nucleolar substrates and regulate related nuclear events, such as regulating the SUMOylation status of TFs. However, although the functional analysis of the SUMO2/3-specific protease, SENP3, has recently been reported (12,28–30), the mechanisms by which the protease functions in response to a variety of cellular stresses in the development of OSCC remain to be elucidated.

Our findings are in agreement with the concept that SENP3 acts as a redox sensor (31). We also demonstrate that SENP3 is redistributed in the subnuclear compartments upon H_2_O_2_ treatment. It is likely that SENP3, preferentially residing in the nucleolus, is continuously degraded in the nucleoplasm in the basal state. Several TF luciferase reporters have been used to investigate whether SENP3 affects transcriptional activity, such as nuclear factor-κB (NF-κB), promyelocytic leukemia protein (PML) and signal transducer and activator of transcription 3 (STAT3); however, the effects have varied (15). This indicates that the de-SUMOylation of TFs by SENP3 is crucial for certain TF activity. In addition, some of these TFs themselves may be indirect substrates of SENP3, which makes the regulation more complex. SENP3 is rapidly stabilised and redistributed from the nucleolus to the nucleoplasm in response to mild oxidative stress, thus serving as a redox sensor. The increased nucleoplasmic SENP3, in turn, acts as an initiator to affect certain TF activity through de-SUMOylation, which may play an important role in the development of OSCC. However, the direct substrates of SENP3 are largely unknown in OSCC. For example, the expression of SUMO-2/3 induces senescence through p53- and pRB-mediated pathways, which can stimulate the transcriptional activity of p53 (19). It remains unknown, however, whether the transcriptional activity of p53 is affected by SENP3.

In conclusion, the present study demonstrates the expression pattern of SENP3 in OSCC and the correlation between SENP3 expression and the differentiation of OSCC. As a redox sensor, SENP3 may play an important role in the course of OSCC tumorigenesis and progression in response to oxidative stress. Further studies are required to clarify the function of overexpression of SENP3 in OSCC and to identify the direct substrates of SENP3. Understanding the molecular mechanisms of OSCC development may provide valuable information on diagnosis and precaution, and may aid in the development of novel therapeutic strategies.

## Figures and Tables

**Table I:** Immunohistochemical evaluation of SENP3 in OSCC patients and oral epithelial tissue adjacent to tumor (Wilcoxon signed-rank test).

		SENP3, no. (%)				
		
Type of tissue	Total	0	1	2	3	P-value
Oral epithelial tissue adjacent to tumor	20	6 (30)	10 (50)	4 (20)	0 (0)	0.02
Cancerous tissue	40	4 (10)	12 (30)	10 (25)	14 (35)	

**Table II:** Correlation between the clinicopathological features and SENP3 expression.

Characteristics	Case no.	SENP3 positivity grade	Non-parametric tests value	P-value
Tobacco smoking				
Yes	17	1.50±0.92	Z=−0.101	0.919
No	23	1.45±0.74		
Alcohol consumption				
Yes	18	2.00±1.41	Z=−0.702	0.472
No	22	1.43±1.74		
Gender				
Male	28	1.59±0.73	Z=−1.294	0.196
Female	12	1.12±0.83		
Tumor site				
Oral cavity	21	1.55±0.82	χ^2^=1.635 d.f.=3	0.652
Gingiva	6	1.25±0.95		
Mouth floor	7	1.33±0.57		
Other	6	1.33±0.57		
Tumor stage				
T1	17	1.66±1.15	χ^2^=1.698 d.f.=3	0.637
T2	12	1.57±0.75		
T3	6	1.50±0.83		
T4	5	1.14±0.69		
Nodal status				
N0	27	1.57±0.85	Z=−0.495	0.621
N1–2	13	1.37±0.71		
Pathological differentiation grade				
Well	23	1.76±0.72	χ^2^=8.014 d.f.=2	0.018
Moderate	13	1.61±0.50		
Poor	4	0.50±0.57		

d.f., degree of freedom.
